# Meal context and food preferences in cancer patients: results from a French self-report survey

**DOI:** 10.1186/s40064-016-2538-1

**Published:** 2016-06-21

**Authors:** Estelle Guerdoux-Ninot, Robert D. Kilgour, Chloé Janiszewski, Marta Jarlier, Jocelyne Meuric, Brigitte Poirée, Solange Buzzo, Grégory Ninot, Julie Courraud, Wendy Wismer, Simon Thezenas, Pierre Senesse

**Affiliations:** SIRIC Montpellier Cancer, Cancer Institute of Montpellier (ICM)-Val d’Aurelle, 208 avenue des Apothicaires, Parc Euromédecine, 34298 Montpellier Cedex 5, France; Epsylon Research Unit EA 4556 Laboratory, Departments of Sport Sciences, Medicine and Psychology, University of Montpellier and University Paul Valery, Rue du Pr. Henri Serre, 34000 Montpellier, France; Department of Exercise Science, The Richard J. Renaud Science Complex, Room SP-165-17, Concordia University, Loyola Campus, 7141 Sherbrooke Street West, Montreal, QC H4B 1R6 Canada; McGill Nutrition and Performance Laboratory (MNUPAL), McGill University Health Centre, Suite 105B, Place Vendome, 5252 de Maisonneuve Ouest, Montreal, QC H4A 3S5 Canada; Department of Clinical Nutrition and Gastroenterology, Cancer Institute of Montpellier (ICM), Val d’Aurelle, 208 avenue des Apothicaires, Parc Euromédecine, 34298 Montpellier Cedex 5, France; Department of Clinical Research, Cancer Institute of Montpellier (ICM), Val d’Aurelle, 208 avenue des Apothicaires, Parc Euromédecine, 34298 Montpellier Cedex 5, France; Biostatistics Unit, Cancer Institute of Montpellier (ICM), Val d’Aurelle, 208 avenue des Apothicaires, Parc Euromédecine, 34298 Montpellier Cedex 5, France; Department of Dietetic and Nutrition, Curie Institute of Paris, 26 rue d’Ulm, 75248 Paris Cedex 05, France; Department of Dietetic, Centre Francois Baclesse of Caen, 3 avenue du Général Harris, BP5026, 14076 Caen, France; Department of Dietetic, Centre Antoine Lacassagne of Nice, 33 Avenue de Valombrose, 06189 Nice Cedex 2, France; Group of Nutrition and Dietary Committees of Anti-Cancer Centers (Interclan CLCC), Villejuif, France; Agricultural, Food and Nutritional Science, University of Alberta, Edmonton, AB T6G 2P5 Canada

**Keywords:** Cancer, Food preferences, Taste, Meal context, Feeding behaviors, Weight loss

## Abstract

**Purpose:**

The present study examined patient self-reports of descriptions, experiences and consequences of meal disturbances and food preferences within a cultural context (i.e., French meal traditions) in various treated cancer patients along their disease trajectory.

**Methods:**

Over 800 questionnaires were sent to 20 cancer treatment centres in France. During a 9-month period, 255 questionnaires were received from five centres. Inclusion criteria included those French patients over 18 years of age, could read and understand French, had an Eastern Cooperative Oncology Group score between 0 and 2, experienced treatment-induced nutrition changes and/or had decreased oral intake. Dietetic staff assessed clinical characteristics while patients completed a 17-item questionnaire.

**Results:**

The majority of patients were diagnosed with breast, gastro-intestinal (GI) tract and head and neck cancers (62 %). Half of the patients (49 %) experienced weight loss >5 %. The main treatment-induced side effects were fatigue, nausea, dry mouth, hypersensitivity to odors and GI tract transit disorders. These discomforts affected eating and drinking in 83 % of patients, inducing appetite loss and selected food aversion. Food preference appeared heterogeneous. Food taste, odor and finally appearance stimulated appetite. Finally, dietary behaviors and satisfaction were driven by the extent to which food was enjoyed.

**Conclusions:**

During oncologic treatments, eating and drinking were affected in more than three-quarters of patients. As recommended by practice guidelines, nutritional assessment and follow-up are required. Personalized nutritional counseling should include the role of the family, patient’s meal traditions, and food habits.

## Background

In disease-free adults, daily food intake is distributed over many meals and snacks. The traditional meal definition in the European culture is a social event that occurs on a regular daily basis at relatively fixed hours, including several dishes presented in succession (Bellisle et al. 2003). Traditionally, French adults are used to ingesting three meals a day. The interval that separates lunch from dinner in the French culture is traditionally interrupted by a small snack that produces long lasting satiety and that accounts for, on average, 18.5 % of the total daily energy intake (Bellisle et al. 2003). However, several factors affect meals in the natural environment, such as social facilitation, subjective hunger, food hedonics, learned habits, and palatability. Food hedonics is one component of the eating and drinking experience and refers to a psychological determination of the extent to which eating and drinking provides feeling of pleasure or displeasure (Boltong et al. 2012). Palatability is defined as the stimulus quality of a substance which determines its acceptability (de Castro et al. 2000). In a study including French participants, higher levels of palatability were found to be related to larger meal portions, meal duration, smaller satiety ratios, greater hunger, and less anxiety/depression (de Castro et al. 2000). Incidentally, palatability has large effects on intake regardless of culture (i.e., for the North American and the French cultures) but appears to be only one of many factors influencing intake (de Castro 2000).

Moreover, several studies have demonstrated an altered food preference in cancer, and food-related quality of life depending on treatment or disease stage (Epstein et al. 2002; Hovan et al. 2010). Patients with head and neck cancers had altered food-related expectations and changes in the “meaning of food” resulting in physical, emotional and social losses (McQuestion et al. 2011). Following curative gastrectomy, patients experienced increased dysphagia, eating restriction, anxiety, taste changes, and body image scores which gradually decreased over 12 months (Kong et al. 2012). Radiotherapy is also well known to impair taste perception during and after cancer treatment (e.g., Ruo Redda and Allis 2006; Irune et al. 2014). Taste changes and food preferences are also expressed differently according to chemotherapy (CT) regimen with significant relationships between ageusia and dry mouth, bitter taste and appetite loss, sour taste and nausea, and anorexia and dry mouth (Zabernigg et al. 2010). As highlighted by recent systematic reviews, the influence of CT changes in food preferences have not been consistently demonstrated (Epstein and Barasch 2010; Boltong and Keast 2012; Gamper et al. 2012). Finally, the extent to which these disturbances play a role in dietary behavior during and after treatment remains relatively unknown. Despite randomized trials of dietary counseling compared to no dietary counseling or to standard practice (e.g., Isenring et al. 2007), there is a lack of information concerning practical management of food intake, particularly meal and food preferences in a cultural context among cancer patients undergoing treatment.

The approach of this study was to explore meal and food preferences among cancer patients within a cultural context (i.e., including meal traditions) because of its pregnant role in feeding behaviors. Therefore, we conducted a multi-centre study to investigate self-reported descriptions and experiences of the meals context and food preferences in cancer patients.

## Methods

### Study population and design

Over a 9-month period, patient self-report surveys were completed anonymously and used to explore patient descriptions, experiences and consequences of meal disturbances.

Height hundred questionnaires were sent from the lead investigation site (Cancer Institute of Montpellier, ICM) to 20 cancer treatment centres in France. Ethical approval to conduct this study and to publish the results was granted from the government through the “Oséo program” Grant. Patients were eligible for inclusion if: (1) they were aged 18 years or over; (2) they were French by culture, were able to read and understand French; (3) they had an Eastern Cooperative Oncology Group (ECOG) Performance Status score of 0, 1 or 2; (4) they had self-reported oral intake changes related to treatment during the dietician clinical assessments; and/or 5) they had an *ingesta* visual analogue scale (*ingesta*-VAS) score <8 (Thibault et al. 2011; Senesse et al. 2014). The *ingesta*-VAS was used for a quick assessment of dietary intake in clinical practice, particularly in patients with weight loss (0 = no ingesta; 10 = usual ingesta). This scale is highly correlated with caloric intake (*p* < 0.0001). Patients defined themselves their level of ingestion of food by ticking a 100-mm line traced on a paper to answer the inquiry “How much do you currently eat on a scale from 0 “*nothing at all*” (far left side of the line) to 10 “*as usual*” (far right side of the line)?” (Thibault et al. 2011).

### Material and procedure

In line with Boltong et al. (2012), the questionnaire was developed by a working group of students, dietitians, medical residents, physicians, and food industry professionals with product development experience. A pilot test of the questionnaire was conducted with 20 patients to ensure item clarity. No change was made to the questionnaire following this test phase.

Dietitians assessed patient demographic and clinical characteristics (e.g., tumor location, anticancer treatment, weight loss), and provided instruction for completion of the questionnaire. The questionnaire consisted of 17 items (Table 1) that related to the description of (1) treatment-related side effects and their impact on eating and drinking; (2) cooking skills; (3) dietary behavior; (4) food preference; (5); experiences with nutrient-enriched food; and (6) preferences for a new dietary product. The questionnaire contained open-ended and multiple-choice questions with an opportunity to provide alternative answers and to choose more than one answer. Two additional questions were about food attributes that stimulate appetite (#4.4) and the advantages that motivate the purchase of new dietary products (#6.1). For these two items, the answers were ranked in descending order of importance on a 7-point and a 9-point scale, respectively (1 = most important).

**Table 1:** Self-report questionnaire^a^

#1 Cancer treatment side effects and their impact on eating and drinking	
#1.1	Describe the side effects induced by the treatment: nausea, swallowing difficulties, mouth ulcers, constipation, dry mouth, chewing difficulties, persistent taste, diarrhea, fatigue, hypersensitivity to odors, vomiting, and/or others.
#1.2	Do these side effects disturb your eating and drinking? Yes or no.
	If yes, describe how: appetite loss, mouth and esophagus pain, fast fullness, food aversion, digestion pain, and/or other.
#1.3	How long do these difficulties persist after treatment?
#2 Cooking skills	
#2.1	Are you able to cook as soon as you come back home? Yes, no or only after *n* days.
#2.2	Are you helped by: a parent/family member/relative, home delivery, ready-to-eat products, and/or others?
#3 Dietary behavior	
#3.1	What meals and/or snacks do you currently have in one day? Breakfast, light morning meal, lunch, light mid-afternoon meal, dinner, and/or other.
#3.2	What is currently your favorite meal? Breakfast, light morning meal, lunch, light mid-afternoon meal, dinner, and/or no preference.
#3.3	What portion would you eat if a full meal was served to you in the hospital or at home? For the first course, for the second course, for the cheese and/or yogurt, for the dessert, and/or for the snack, would you eat 0, 1/4, 1/2, 3/4, 1 or 2 portions?
#4 Food preferences	
#4.1	What do you currently prefer to eat: salty, sweet, hot, cold, into pieces, minced, blended, creamy, liquid, and/or other?
#4.2	Since the beginning of the treatment, what food, salty or sweet courses do you prefer to eat?
#4.3	Specify for what foods you have developed nausea or an aversion.
#4.4	What are the most important food attributes that stimulate your appetite (in descending order of importance from 1 to 7): taste, aspect, odor, consistency, quantity, presentation, and/or other?
#4.5	What type of snacks do you prefer to eat: salty, sweet, creamy desserts, ice creams, biscuits, milky beverages, others beverages, and/or others?
#5 Nutrient-enriched foods	
#5.1	Have you already experimented with enriched or fortified foods? Yes or no.
	If yes, in what context have you experimented with it: in the hospital, at home, and/or, other?
	If yes, please specify: blended courses, creamy desserts, milky beverages, non-milky beverages, and/or others?
	If yes, are you satisfied with the products: very satisfied, satisfied or not satisfied? Please specify why.
#6 Preferences for a new dietary product	
#6.1	If new dietary products were developed, what advantages would motivate you to purchase them (in descending order of importance from 1 to 9): taste, sale price, ready-to-eat, dietary counseling, medical prescription, mode of preservation, nutritional value, partial reimbursement, or others?
#6.2	Where do you prefer to find these new products: pharmacies, dietary shops, supermarkets, and/or home delivery?
#6.3	What type of new products would you prefer: frozen, canned, fresh or other?

^a^Translated from French to English

Patients’ written informed consent was obtained prior to the completion of the questionnaire. Institutional review boards of each investigational site approved the study protocol. Questionnaires were returned to the ICM for data analysis.

### Statistical considerations

Categorical variables were reported by means of frequency and percentages. For continuous variables, medians, means, standard deviations and range values were computed. The association between demographic, clinical characteristics and different items included in the self-report questionnaire was assessed using Pearson’s Chi square test or Fisher’s exact test when applicable for categorical variables, and using Kruskal–Wallis or Student *T* test for continuous variables.

All reported p-values are two-sided and were considered significant at the 5 % level. Data from open-ended questions were handled and analysed with NVivo10 software, which usually supports qualitative methods research. In the current study, they were explored using descriptive content analysis. Themes are only reported with quotes considered to be typical unless explicitly noted (Sandelowski 2010). Only brief comments from the qualitative analysis are reported. Statistical analysis was performed using STATA v.11.0 software (Stata Corporation, College Station, TX, USA).

## Results

### Patient sample

From the 800 distributed questionnaires, 255 were returned (31.8 %) from the 20 oncology-specialized hospitals, in particular from the ICM, the Francois Baclesse Center of Caen, the Curie Institute of Paris, the Paoli Calmettes Institute of Marseille, and the Antoine Lacassagne Center of Nice. Patients completed the questionnaires as outpatients (52.1 %), or inpatients (full-time hospitalization, 47.8 %). Patient characteristics are shown in Table 2. Patients were on average 59.5 years old (±13 years, SD), ranging from 22 to 89 years. All of them were French by culture and adopted French meal traditions. Most were women (60.0 %), aged from 50 to 69 years (56.4 %), lived as a couple (69.8 %), had one or two children (64.4 %) and did not work (48.2 %) or were on sick leave (30.2 %). Tumors were localized mostly in breast (27.1 %), GI tract (22.0 %) and head and neck (12.9 %). Most patients received a single round of CT (71.4 %). Most patients had altered functional abilities; 39.2 % of patients were restricted in their ability to conduct physically strenuous activities but were ambulatory and able to carry out work of a light or sedentary nature (ECOG performance status score of 1), and 28.2 % were ambulatory and capable of all self-care but unable to carry out any work activities (ECOG score of 2). Half of patients (50.4 %) suffered from weight loss ≥5 % compared with their pre-treatment weight (Fearon et al. 2011). Insufficient caloric intake, as defined by a score <8 at the *ingesta* visual analogue scale, was reported in 54.1 % of cases. Dietitians noticed that two-thirds of patients suffering from weight loss ≥5 % (65.7 %) consumed sip feeds, 48.0 % split meals and 15.8 % had artificial nutrition. Side effects of cancer treatment impacting oral intake are detailed in Table 3.

**Table 2:** Patient demographic and clinical characteristics (*n* = 255)

Characteristic	*n* (%)
Age	
<60 years	111 (43.5)
≥60 years	144 (56.5)
Female sex	153 (60.0)
ECOG performance status^a^	
0	76 (29.8)
1	100 (39.2)
2	72 (28.2)
Side effects on oral intake	244 (95.7)
*Ingesta* visual analogue scale^b^	
(0–6)	90 (35.3)
(7 and 8)	48 (18.8)
(8–10)	100 (39.2)
Artificial nutrition	38 (15.8)
Personal and professional status	
Live in southern France	143 (56.1)
As a couple	178 (69.8)
Active^c^	27 (10.6)
Inactive^d^	123 (48.2)
Sick leave	77 (30.2)
Tumor location	
Breast	69 (27.1)
Digestive system	56 (22.0)
Head and neck	33 (12.9)
Hematopoietic system	18 (7.1)
Lung	17 (6.7)
Gynecology	17 (6.7)
Others	35 (13.7)
Anticancer treatment	
Chemotherapy	182 (71.4)
Radiotherapy	7 (2.7)
Chemotherapy + radiotherapy	26 (10.2)
Surgery	23 (9.0)
Others (e.g., antibiotic therapy)	11 (4.3)
Weight loss (compared with baseline) (%)	
<5	121 (47.5)
5–10	59 (23.1)
10–20	53 (20.8)
≥20	13 (5.1)

^a^The Eastern Cooperative Oncology Group (ECOG) performance status refers to functional ability scores (ranged from 0 to 5) to quantify cancer patients general well-being and activities of daily life

^b^The ingesta visual/verbal analogue scale is used for a quick assessment of dietary intake in clinical practice, particularly in patients with weight loss (0 = no ingesta; 10 = usual ingesta). This scale is highly correlated with caloric intake. Patients defined themselves their level of ingestion of food between 0 (nothing) and 10 (as usual)

^c^Patients were considered to be active if they had a job

^d^Patients were considered to be inactive if they had no job or were retired

**Table 3:** Treatment side effects

Type of side effects (*n* = 255)	*n* (%)
Fatigue	190 (74.5)
Nausea	141 (55.3)
Dry mouth	124 (48.6)
Hypersensitivity to odors	91 (35.7)
Constipation	87 (34.1)
Diarrhea	83 (32.5)
Mouth ulcers	64 (25.1)
Swallowing difficulties	62 (24.3)
Vomiting	55 (21.6)
Persistent taste	36 (14.1)
Chewing difficulties	35 (13.7)
Others (e.g., dysgeusia)	52 (20.4)

### Responses to the themes of the self-report questionnaire

#### #1 Treatment-related side effects and impact on eating and drinking

The main side effects induced by the treatment therapies were fatigue (74.5 %), nausea (55.3 %), dry mouth (48.6 %), hypersensitivity to odors (35.7 %) and constipation (34.1 %). Most patients (54.5 %) reported digestive tract disorders such as constipation or diarrhea (Table 3). Treatment-related side effects affected eating and drinking in 83.1 % of patients, principally inducing appetite loss (66.8 % of patients who reported an impact on eating and drinking), food aversion (51.4 %) and satiety (39.7 %) (Table 4). These disturbances on eating and drinking persisted for 10.8 days on average after treatment (ranging from 1 to 120 days). From open questions in the questionnaires, we also identified patient-related theme of feeling disturbed by the side effects. The strongest message from patients was that they experienced taste modifications such as “*I have no taste anymore*” (centers of Marseille and Caen), “*I have lost taste*” (center of Montpellier), “*my taste has been modified*” (center of Nice), “*I have strange taste in my mouth*” (center of Paris). They also qualitatively reported a loss of pleasure described as “*everything considerate, I feel a discomfort*” or explicitly as “*I have lost pleasure when eating and drinking*”.

**Table 4:** Side effects and oral intake impact

Type of perturbation of oral intake (*n* = 212)	*n* (%)
Appetite loss	143 (66.8)
Food aversion	110 (51.4)
Satiety	85 (39.7)
Mouth pain	54 (25.2)
Abdominal pain during digestion	26 (12.1)
Others (e.g., ageusia)	23 (10.7)

#### #2 Cooking skills

Over half the patients (53.3 %) were able to cook as soon as they returned home from a hospital day or from a treatment session (i.e., after a hospital stay), and 25.9 % started cooking again after 4–5 days. Patients’ parents were the main source of help (84.7 %) irrespective of the patients’ familial situation (i.e., as a couple or not).

#### #3 Dietary behavior

Patients reported to have daily dinner (91.8 %), breakfast (90.2 %) and lunch (88.2 %). Outpatients (47.9 % of patients) had more lunch meals than inpatients (91.8 vs. 83.0 %, *p* = 0.042) and tended to have more dinners (95.1 vs. 88.4 %, *p* = 0.061). However, breakfast was identified as the favorite meal for 55.7 % of patients when compared with lunch and dinner (29.8 % and 20.4 %, respectively), whether they were an inpatient or outpatient.

A typical French meal consists of 4 courses: typically the “first course” (e.g., salad) is followed by the “second course” (e.g., meat with vegetables), then completed by some “cheese and/or yogurt”, and finally by a “dessert” (e.g., fruit or cake). Regarding the portion that patients would eat if a full French meal was served in the hospital or at home, 29.4 % declared that they did not eat a first course. Conversely, 36.5 % reported they would eat one portion of the second course and 23.9 % would eat half a portion. Most patients (58.4 %) would eat one portion of cheese and/or yogurt, and 56.1 % thought they would eat one dessert portion. Similarly, a typical French snack (around 11 a.m. or 4 p.m.) consists of fruit or cake with a tea or coffee. Over half (57.3 %) the patients declined to snack.

Furthermore, results showed a significant difference between the type of hospitalization and the consumption of the first and second courses. When compared with inpatients, a significantly higher percentage of outpatients declared to eat one entire portion or more of the first courses (33.3 vs. 20.0 %, *p* = 0.024) and second courses (47.9 vs. 24.6 %, *p* < 0.001).

Finally, data from open-ended question were mainly linked to a reduction of the usual eaten portions. A patient explained for example that “*during five days, I* [he] *don’t eat afternoon snacks anymore… and I* [he] *don’t eat much…*” while another reported that “*I* [he] *don’t eat anymore during the chemotherapy*” or “*I must eat very light because all that is solid hurts my esophagus*”.

#### #4 Food preferences

Most patients preferred salty foods (57.6 %) rather than sweet ones (39.6 %, *p* < 0.001) in general. They reported a preference for hot (58.7 %) rather than cold food (29.8 %), *p* < 0.001). Concerning the textures, food cut into pieces was preferred by 27.8 % of patients, followed by creamy (20.8 %), liquid (14.1 %), minced (13.7 %) and lastly by blended food (9.8 %). However, sweet foods were as valued as salty foods when patients detailed their preferences. Qualitatively, the strongest message from patients was that the seasoning level drove their preferences, for example “*I prefer foods that are spicy*”, “*foods that are peppery*” or conversely “*I eat strictly without salt*” *or* “*I like dishes that are not too much salty nor too much sweet*”. Results about the descriptive preferences are then reported as percentages of responses in Table 5. The most enjoyed foods were fruits (49 %, including compotes), dairy products (45 %), and after, pasta, and red meat (40 %). On the other hand, more than two-thirds of patients (69 %) reported an aversion to specific foods, particularly red meat (15.4 %) and meals with sauce (10.9 %). The key theme identified was the preference for the typical French courses. It included French specialties (e.g., “*quiche*”, “*bread*”) and also the Mediterranean diet, incorporating a majority of “*fruits and vegetables*”, “*fish*” *and* “*soup*”.

**Table 5:** Food preferences and aversions in cancer patients

	*n* (%)
Main preferences (*n* = 205)	
Fruits	100 (48.8)
Dairy products	92 (44.9)
Pasta	86 (42.0)
Red meat	82 (40.0)
Mashed potatoes	74 (36.1)
Vegetables	74 (36.1)
Soup	68 (33.2)
Fish	62 (30.2)
White meat	42 (20.5)
Milky dessert	40 (19.5)
Rice	26 (12.7)
Salad	20 (9.8)
Main food aversions (*n* = 175)	
Red meat	27 (15.4)
Meals and meat with sauce	19 (10.9)
Everything	13 (7.4)
Sweet	9 (5.1)
Chocolate	7 (4.0)
Cheese	7 (4.0)
Coffee	7 (4.0)

**Fig. 1:**
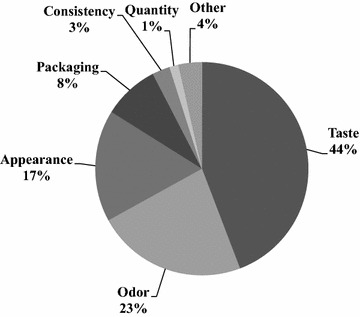
Percentage of patients who chose a characteristic (among taste, appearance, odor, consistency, quantity, packaging and other) as the most important sensory quality that stimulates appetite (*n* = 255)

Patients then ranked a list of the most important attributes stimulating appetite (i.e., taste, appearance, odor, texture, quantity, packaging and other) in descending order of importance on a 7-point scale (1 = most important). Taste was the most frequently cited quality to stimulate appetite (mean score of 2.4), followed by odor and appearance (both 3.0), presentation (4.1), consistency (4.3) and finally quantity (4.6) (Fig. 1). Some patients added qualitatively that the temperature was as another important attribute that stimulated appetite.

Concerning snacks, less than half the patients did not report any taste (41.6 %) and snack type preference (47.5 %). However, patients indicating a taste preference preferred sweet snacks (46.7 %) rather than salty ones (15.7 %, *p* = 0.003) with no significant difference among biscuits (32.5 %), creamy desserts (16.5 %) and ice cream (20.4 %, *p* = 0.34). In summary, most of patients had reported a preference for salty meals but for sweet snacks. Interestingly, patients who lived in southern France (i.e., 56.1 % lived in Nice, Marseille or Montpellier) had a greater preference for ice creams compared with patients living in the north (26.6 vs. 12.5 %, *p* < 0.05). No other difference was found in responses from the diverse geographical areas. Milky beverages (17.6 %) were preferred less than other beverages (38.0 %) such as tea, coffee or herbal tea (without stimulant) and fruit juices (*p* = 0.002). Finally, answers from open-ended question focused mostly on “*fruits*” as another potential snack.

#### #5 Nutrient-enriched foods

Fifty-four percent of patients (*n* = 129) had already experimented with enriched or fortified foods, 43.4 % at home, 36.4 % in the hospital, 19.4 % at both places. Oral nutritional supplements were mostly milky beverages (69.8 %), creamy desserts (29.5 %), and non-milky drinks (24.0 %). From the open-ended question, most patients reported to have already eaten hyperprotein soup. More than two-thirds of patients (70.5 %) were satisfied or very satisfied with the products. Among those who expressed dissatisfaction with the nutrient-enriched foods, the majority (84 %) identified taste as the major cause of their dissatisfaction. Qualitative data enlighten that the products were mostly liked because they were “*easy to digest*” while they were mostly disliked because of their “*bad, viscous or stodgy taste*”.

#### #6 Preferences for a new dietary product

Of the characteristics that ranked the motivation of purchasing a new dietary product (from 1 to 9, where 1 = most important), taste was considered to be the most important (mean score of 2.4), followed by nutritional value, dietary counseling (both 3.8), and medical prescription (4.4). Patients expressed a desire to find these new products in supermarkets (59.6 %), community pharmacies (29.0 %) and dietary shops (20.4 %). Home delivery would also be appreciated by 17.3 % of patients. Preference for product form was for fresh (74.1 %), frozen (25.9 %) and canned products (17.3 %). The other themes that patients freely expressed were the visual aspect of the product (e.g., “*presentation, color, appearance*”) and its “*organic*” character.

## Discussion

This is one of the first studies to describe meal-related behaviors and food preferences of cancer patients within a cultural context (i.e., considering French meal traditions). We used patient self-reports to investigate descriptions and experiences of the meal situation focusing on symptoms, behaviors, and changes. The majority of patients had a good ECOG performance status (about 70 % were grade 0 or 1), and more than 90 % received an oncologic treatment. Indeed, the first remarkable result was that treatment-related side effects affected eating and drinking in 83.1 % of cases irrespective of tumor location and of type of treatment.

In fact, the main consequence of altered eating and drinking was decreased food and energy intake. In our study, more than 50 % of patients suffered weight loss ≥5 %, increasing their risk of potential health complications (Dewys et al. 1980; Andreyev et al. 1998; Bellisle 2003; Argilés 2005; Arends et al. 2006; Senesse et al. 2014). Most patients had breast or GI tract cancer treated with chemotherapy. Indeed, the main treatment-induced side effects included fatigue, nausea, dry mouth, altered sensations to smell and digestive transit disorders. In line with previous studies (Zabernigg et al. 2010; Coa et al. 2015), these side effects affected eating and drinking in 83 % of patients, by inducing principally appetite loss and aversion to specific foods. Due to the variety of symptoms affecting patients, it is essential to provide a comprehensive approach integrating dietary advice and other specialists such as speech language pathologists, occupational therapists or social workers, depending on the disease and treatment (Senesse et al. 2014). Further studies showed that dietary counseling reduced weight loss, improved quality of life, and reduced toxicity in irradiated patients with head and neck, and gastrointestinal cancer (Ravasco et al. 2005a, b; Isenring et al. 2012). Dietary counseling involving the prescription of a therapeutically adjusted diet should be more widely provided to such patients. Nutritional guidelines highlight the importance of dietary counseling; however, there is a lack information concerning the definition of dietary counseling used to demonstrate its benefits (Boltong et al. 2012).

Dietary counseling must include meal and food preferences within the cultural context and the patient’s home situation. Three out of 4 patients were concerned with fatigue and were not able to resume cooking and meal preparation immediately after treatment. Patients’ parents were the main source of help even if the patient was as a couple. Hence, dietary counseling should also involve them, and more broadly the family. Then, cancer-related fatigue is well known to be one of the most prevalent and burdensome symptoms (Servaes et al. 2002; Tomlinson et al. 2013) and often becomes a major distressing symptom (Minton et al. 2013). The development of a specific physical activity intervention could reduce asthenia and fatigue, and may stimulate appetite thus leading to an increase in oral intake (Bortolon et al. 2014). Concerning meal-related behaviors and cultural context, more than 50 % of patients identified breakfast as their favorite meal. Patients also reported a preference for lunch and dinner at home rather than in the hospital, eating salty and hot meals, and food cut into pieces. In fact, our results highlight the importance of meals and hedonics food (i.e., the extent to which eating and drinking provides affect of pleasure or displeasure) for French oncologic patients, also documented among healthy adults in France (de Castro et al. 2000; Bellisle et al. 2003). In this study, meals (i.e., breakfast, lunch and dinner) provided the greatest contribution to food intake, while also demonstrating a specific role for snacks. The consumption of snacks would appear to be a simple way to increase energy and protein intake. For example, almost half of all patients ate at least one portion of cheese and/or yogurt, and also one dessert portion with ice cream preference in the southern region of the country.

Oncologic treatment effects may differentially affect food preference as taste quality changes have been shown to be individual (Brisbois et al. 2011). Food preference information compiled from six studies indicates that caffeinated foods and drinks, red meat and citrus fruit or juices are more likely to become an aversion during CT than other foods or beverages (Boltong and Keast 2012). In a recent study among hospitalised haematological cancer patients, fresh fruit, ice cream, cheese and mashed potatoes with bacon were the most desired food items (Okkels et al. 2016). Our results were in agreement with a recent qualitative study in which patients expressed a reduced intensity of elements of flavor (taste “fading” or “blending out”) (Coa et al. 2015; Okkels et al. 2016). Food hedonics are broader than the eating and drinking experience (Boltong and Keast 2012). They may include a good palatability and a psychological determination of the extent to which diet provides emotion of pleasure or displeasure. For example, in our sample, some patients reported enjoyment of fruit, dairy products and red meat, whereas these same foods repulsed others. Finally, qualitative data enlighten the importance of cultural habits: beyond the treatment-induced side effects, patients reported preferences for the typical French courses, in particular for the “Mediterranean diet”.

Participants indicated that taste was the most important attribute stimulating appetite, and also the most important benefit rewarding the purchase of a new dietary product after the nutritional value itself. Also, odor and appearance seemed to influence appetite more than the quantity itself. Interestedly, a new cognitive propriety that may also influence appetite has emerged from the qualitative data: whether the food was organic or not. First, our results confirmed that there are many factors that affect meals in cancer context, as it was shown in the natural environment (de Castro et al. 2000). Palatability had only a limited influence on intake. Then, it is important to note that taste is often confused with flavors (Spence 2013). Taste perception refers to those sensations that are elicited by the stimulation of the gustatory receptors on the tongue (sweet, sour, salty, bitter and more recently, umami—savory) (Stevenson 2009). However, people virtually never experience pure isolated taste and mostly experience flavors, resulting from the combination of taste, retronasal olfaction and trigeminal inputs (Small 2012). “*Fruity*”, “*meaty*”, “*floral*” and “*burnt*” are all flavor descriptions (Verhagen 2007). Actually, flavor is perhaps the most multimodal of all of our sensory experiences (Auvray and Spence 2008). Therefore, it is now timely to suggest that cognitive neuroscientists integrate the study of multisensory flavor perception in the oncology field. As it was demonstrated with visual–auditory stimuli in older adults (Laurienti et al. 2006), the use of multiple sensory channels may represent an effective compensatory strategy to overcome the unisensory deficits induced by cancer treatment. There is hope that our growing psychological scientific understanding of multisensory flavor may help the food industries to deliver adjusted and more personalized products for better health care.

In summary, given their impact on morbidity, mortality and quality of life (Zabernigg et al. 2010), taste and/or smell changes and their impact on food hedonics should be considered very seriously in clinical practice. Such data should be integrated in tools assessing quality of life and should be used to improve nutritional status (Hutton et al. 2007).

Finally, we identified essential and practical advice for health-care professionals in order to improve energy intake and to prevent significant weight loss before and during treatment therapy.

- Eating and drinking are affected in most of cases during treatment. A systematic nutritional assessment is required as well as a follow-up as recommended in guidelines (Arends et al. 2006; August et al. 2009; Senesse et al. 2014).
- Focusing dietary counseling on meals, especially on breakfast, should be efficient to increase dietary intake addressed to in- and outpatients.
- At home, dietary counseling should also involve patients’ parents who are the main source of help, and more broadly should involve the family. Therefore, lunch and dinner could be improved easily.
- Prescription of a therapeutic diet should be personalized and adjusted to the patients’ food preferences and meal-related behaviors, including patient’s culture (in terms of meal traditions), geographical area, and habits.

Additional investigations, including both qualitative and quantitative methods (i.e., mixed approaches), are necessary to further reduce the gap between self-report experiences and evidence-based strategies.

We have identified several limitations to our study. The questionnaire used to assess dietary changes may have been too long and complex for some patients who required the help of the oncology dietitian staff for its completion. Even if there were no biases with interpretation and responses by participants who required some help (it concerned only a few patients), it made heavy the procedure for those participants. Also, it did not clearly ask patients about the need for counseling and nutritional support, nor whether participants presently received this service. No information was given about the average length of time these patients have been receiving treatment. Our results should be interpreted and generalized with caution. These concerns relate specifically to the relatively low response rate and heterogeneity of the sample. Our study population was purposely heterogeneous, including patients with different types, locations, and stages of cancer, and also different therapies. While published studies usually focus on a precise disease and treatment, we aimed to reflect the diversity of patients met in real practice.

In conclusion, this study showed a high prevalence of symptoms reducing appetite and changes to meal and food preference during oncologic treatment. Individual dietary counseling has been shown to reduce morbidity and mortality, and to improve functional performance and well-being (Bauer 2007; Meuric and Besnard 2012). However international guidelines addressing dietary counseling should incorporate meal and food preferences in the cultural context specific to each country (i.e. meal traditions).

## Abbreviations

GI: gastro-intestinal
CT: chemotherapy
ECOG: Eastern Cooperative Oncology Group
ICM: Cancer Institute of Montpellier
*ingesta*-VAS: *ingesta* visual/verbal analogue scale
